# Facile fabricating of rGO and Au/rGO nanocomposites using Brassica oleracea var. gongylodes biomass for non-invasive approach in cancer therapy

**DOI:** 10.1038/s41598-021-91352-7

**Published:** 2021-06-07

**Authors:** Fatemeh Yousefimehr, Saeed Jafarirad, Roya Salehi, Mohammad Sadegh Zakerhamidi

**Affiliations:** 1 Research Institute of Bioscience and Biotechnology, University of Tabriz, Tabriz, Iran; 2 Department of Organic and Biochemistry, Faculty of Chemistry, University of Tabriz, Tabriz, Iran; 3 Drug Applied Research Center, and Department of Medical Nanotechnology, Faculty of Advanced Medical Science, Tabriz University of Medical Science, Tabriz, Iran; 4 Faculty of Physics, University of Tabriz, Tabriz, Iran; 5 Research Institute for Applied Physics and Astronomy, University of Tabriz, Tabriz, Iran

**Keywords:** Biochemistry, Chemical biology, Chemistry, Materials science, Physics

## Abstract

In this study, we report a facile green-synthesis route for the fabrication of reduced graphene oxide (rGO) using biomass of Brassica oleracea var. gongylodes (B. oleracea). In addition, we have attempted to provide a green synthesis approach to prepare Gold nanoparticles (Au NPs) on the surface of rGO by using stem extract of B. oleracea. The synthesized Au/rGO nanocomposite was evaluated using UV–visible and FTIR spectroscopy, XRD, Raman, FE-SEM, EDX, AFM and DLS techniques. The obtained results demonstrated that the synthesized Au NPs on the surface of rGO was spherical with sizes ranging about 12–18 nm. The Au/rGO NC was, also, developed as photo-synthesizer system for the photothermal therapy (PTT) of MCF7 breast cancer cells. The near-infrared (NIR) photothermal properties of Au/rGO NCs was evaluated using a continuous laser at 808 nm with power densities of 1 W.cm^−2^. Their photothermal efficacy on MCF7 breast cancer cells after optimizing the proper concentration of the NCs were evaluated by MTT assay, Cell cycle and DAPI staining. In addition, the potential of the synthesized Au/rGO NCs on reactive oxygen species generating and antioxidant activity were assessed by DPPH. Au/rGO NCs possess high capacity to light-to-heat conversion for absorption in range NIR light, and it is able to therapeutic effects on MCF7 cells at a low concentration. The maximum amount of cell death is 40.12% which was observed in treatment groups that received a combination of Au/rGO NCs and laser irradiation. The results demonstrate that the nanomaterials synthesized by green approach lead to efficient destruction of cancer cell and might thus serve as an excellent theranostic agent in Photothermal therapy applications.

## Introduction

Disease prevalently "Breast cancer" which the most rampant cause of cancer death and the most among women^1^. In spite of great efforts and investigations that have been made to treat cancer within past decades, the lack of an effective method is still a great challenge in clinical applications^2,3^. Recently novel cancer therapy methods have been developed extensively based on nanotechnology as an effective technique of advanced-stage cancers. Nonstop progress in the methods for breast cancer treatment, understanding the associated mechanisms, and the improvement of novel medicines will consequence in more effective cancer treatment. Photothermal therapy (PTT) is a non-invasive cancer therapy method compared to traditionally cancer treatment methods like radiotherapy and chemotherapy. In PTT method tumor tissue is exposed to light and the absorbed light is transformed to heat to stimulate the tumor destruction^1^. The extensive application of PTT in clinical cancer therapy was limited due to the incomplete penetration of NIR irradiation in tissues which lead to inadequate ablation of large tumors and also small tumors placed inside the body^4^. This problem is largely resolved by using high-power NIR irradiation (around 2 W.cm^-2^ for rGO nanostructures)^5^. Though, the high power may damage nearby normal tissues mostly, in extended NIR irradiation exposure^6,7^. Light-absorbing nanomaterials which have optical absorption in the near-infrared are used to stimulate photothermal destruction of tumor cells^6^. GO as a hydrophilic lamellar material with a 2D structure, has earned exceptional attention in biomedicine because of its excellent biocompatibility, large surface area and ignorable toxicity^8^. Numerous nanostructures, like Au NPs, GO, rGO, carbon nanotubes and iron oxide nanoparticles have been proven that show photothermal effects and used for the hyper-thermic therapy of cancer^8–10^. On the other hand, the modified graphene-based nanostructures were stable, biocompatible and exhibited higher solubility in water and biological fluids. The use of novel Au NPs with a more efficient light-to-heat conversion is the other option^11,12^. The embedding and coating of Au NPs within GO and rGO could be a way into this direction^13,14^. Therefore, synergistic properties of both Au NPs and rGO will offer increased potential for applications in biomedicine. Au NPs are currently considered to be the most appropriate nanoparticles for cancer therapy^8,9^. Photoexcitation of Au NPs at their surface plasmon resonance band can efficiently convert photon energy into heat and can be used for the photothermal ablation of cancer cells^14^. Hence the functionalization of graphene-based nanostructures with Zero-dimensional Au NPs into a hybrid structure can fabricate Au/rGO NCs. Au/rGO NCs, also, indicated remarkable performance in different fields of applications such as surface-enhanced Raman scattering^15^, biosensors^16^, catalysis^17^, antimicrobial activity against bacteria^18^.

Graphene-based materials have been produced via chemical reduction reaction of graphite^19,20^ and high-temperature annealing reduction^21^. However, fabrication of graphene through an easy route has remained a challenging issue so far^22^. Recently, several methods for green and facile production of Au/rGO NCs using green reagents such as chitosan^23^, ascorbic acid^24^, sodium citrate^25^, and tyrosine^26^ were reported. Furthermore, plant biomasses demonstrate to be an outstanding precursor for the eco-friendly fabrication of carbon nanostructures since they possess considerable amounts of carbon elements that can be used for generating carbon nanostructures.

The *B. oleracea* extract reveals the presence of many phytochemicals, such as glycosides, alkaloids, saponnins, flavonoids, tannins, steroids, terpenes^27–30^. In addition, the glucosinolate family is secondary metabolites that are the most abundant in the *B. oleracea* plant and are rich sources of sulfur. Isolated phyto-compounds from this vegetable have very important biological activities including cardioprotective, anti-diabetic, antioxidant, anti-cancer, hypolipidemic and antihyperglycemic^31^.

Herein, we introduce a facile one-pot route for the generation of rGO using biomass of *B. oleracea*. Thus, *B. oleracea* biomass would exploit as a source of carbon for synthesizing rGO. Moreover, we utilized *B. oleracea* stem extract as a non-toxic green reductant and stabilizing reagent in the production of Au/rGO NCs as a novel biocompatible photosynthesizer. To the best of our knowledge, no work has been reported on the PTT application of Au/rGO NCs which is prepared by using *B. oleracea* biomass and stem extract. We believe that such nanostructures can open up new perspectives in cancer treatments.

## Materials and methods

### Material

The stems of B. oleracea were collected from Botanical Garden of Tabriz University. MCF7 breast cancer cell line was purchased from the Pasteur Institute of Iran. Fetal bovine serum (FBS), trypsin, Roswell park memorial institute 1640 growth medium (RPMI) and penicillin/streptomycin were purchased from Gibco. Dimethyl sulphoxide (DMSO) and ethanol 96% were purchased from Merck. 2,2-diphenyl-1-picrylhydrazyl (DPPH), (3-[4,5-dimethylthiazol-2-yl]-2,5 diphenyltetrazolium bromide) (MTT), (4′,6-diamidino-2-phenylindole) (DAPI), Propidium iodide, amphotericin B, Ribonoclase A, and Au(III) chloride hydrate were obtained from sigma Aldrich companies.

### Instruments and reagents

UV–Vis spectra of the rGO and Au/rGO NCs were recorded using Hitachi, U-2900 UV–Vis in the range of 200–800 nm. Fourier transform infrared (FTIR) spectra were recorded using TENSOR 27-Bruker in the range of 400–4000 cm^−1^. Crystal structures of rGO and Au/rGO NCs were characterized by X-Ray Diffraction technique (XRD) (D500, Siemens Diffractometer-Germany)^32^. This diffractometer was configured with a graphite monochrometer, CuKα radiation at accelerating voltage of 40 kV/30 mA, over the range 10–80° 2θ, step size 0.02° and 60 s count time. Morphology, size and the materials percentage were performed by using a Field Emission-Scanning electron microscopy (SEM) and energy-dispersive X-ray spectrometry (EDX) analysis MIRA3 FE-SEM, Tescan Co, Brno, Czech Republic. This microscope has a LVSTD (Low Vacuum Secondary Electron Tescan Detector) at voltage of 30 kV. The samples were dispersed on an aluminum substrate. Then, the dispersed particles were treated by gold coat onto the sample containing substrate. Atomic Force Microscopy (AFM) of Nanosurf Mobile, (Nanosurf Switzerland) was used to evaluate the samples. In addition, the size, polydispersity index (PDI) and zeta potential were identified using Dynamic Light Scattering (DLS) Malvern-UK. Deionized water was used as dispersant and scattering angle of 90j was used for DLS evaluations at *k* = 632.8 nm. Prior to measurement, the samples were ultra-sonicated in sonication bath (Bandelin Sononrex Digitec, 40 kHz). Temperature was set to 25 °C for 20 Min and the samples were left for 60 Min before measurements. Ratio of the intensity of D- Raman peak and G- Raman peak (ID/IG) was measured by using TakRam, Teksan Co, Iran (532 nm of Nd:YAG laser in the range of 4600–100).

### Extraction of *B. oleracea* extract

The fresh *B. oleracea*, as field grown plant, was collected from the Botanical Garden of Tabriz University and identified in herbarium of Tabriz University of Medical Sciences (East Azarbaijan, Tabriz, Iran). B. *oleracea* is not a wild plant and its collection for research goals from the Botanical Garden of Tabriz University was according to institutional rules of the university and did not need any permission or license. The collected plant washed with deionized water, sliced into a slim layer and reserved in dark to dry completely. Slices of *B. oleracea* was dusted and mixed in deionized water for 24 h. Then the obtained extract was ultrasonicated (180 W, 2 min) and filtered.

### Green synthesis of rGO

*Biomasses of B. oleracea* stem was used as a source of carbon for the preparation of rGO. The dried and compressed biomass was powdered. Then, 2 g of powder was put in the microwave (900 W, 30 min)^33^. A change of color from cream to dark brown indicated that the process was completed. Thereafter, the obtained powder dissolved in 20 ml deionized water and transmitted to teflon-lined stainless steel autoclave, which was put in a programmable oven. The autoclave was sealed and heated at 180 °C for 15 h and then allowed to cool to room temperature. The products were filtered off, washed several times with deionized water, and finally dried in a vacuum at 70 °C for 4 h.

### Green synthesis of Au/rGO NC

One-pot synthesis of Au/rGO NCs was performed in the presence 0.1 g Au(III) chloride hydrate salt, 1 g rGO and 20 mL of *B. oleracea* extract^34^. Components moved into teflon-lined stainless steel autoclave and heated at 140 °C for 5 h and then allowed to cool to room temperature. After that, the obtained mixture was ultrasonicated for 30 min. The obtained dispersion was filtered, eluted with deionized water and ethanol and finally dried at 50 °C.

### In vitro ROS Determination with 2,2-diphenyl-1-picrylhydrazyl (DPPH) assay

The amount of ROS and other free radicals of Au/rGO NC were investigated based on our previous literature^34^.

### Cellular uptake of Au/rGO NCs

Cellular uptake assay was performed in order to determine the permeability of nanoparticles into the MCF7 cell line. Au/rGO NCs were marked by an aqueous solution with a ratio of 1:200 from rhodamine-B to Au/rGO NCs and was stirred overnight under dark condition. Afterwards, the rhodamine-B labelled NCs were separated from free rhodamine-B by repeated washing and centrifuging steps. MCF7 cells at the density of 2 × 10^5^ cells per well were seeded in 6 well plates containing 12 mm coverslips and incubated for 24 h and then treated with a defined concentration of rhodamine-B labelled NCs. After 0.5 h, 1.5 h and 3 h residual NCs was removed and washed with PBS and rhodamine-B labelled NCs uptakes were observed using a fluorescence microscope (Olympus microscope Bh2- FCA, Japan).

### Cell viability assay

MCF7 cell lines were grown in complete medium containing RPMI 1640 with 10% Fetal bovine serum (FBS), 0.05 mg/mL penicillin, 0.08 mg/mL streptomycin and atmospheric conditions of 37℃, 5% CO_2_, and 95% humidity. Then after reaching confluency, cells were seeded in 96-well culture plates, and after complete attachment of cells on plates, cells were treated with different concentrations of Au/rGO NCs and further incubated for 24 h. Untreated cells were considered as control. Then cell viability was evaluated with MTT assay method as described previously^9^.

### Laser therapy

MCF7 cells were seeded in a 96 well cell culture plate (1 × 10^4^ cells per well in a 96-well plate) and incubated for 24 h at 37 °C in a humidified 5% CO_2_ atmosphere. Au/rGO NCs with concentration of (12.5 µg/mL) was incubated with MCF7 cells. After 3 h of incubation, the culture medium containing un-uptake NCs was removed and a fresh medium was added and incubated for 24 h in a 5% CO_2_, 95% air humidified incubator at 37 °C. After this time of incubation, the culture medium was removed and 200 µL fresh medium was added. The cells were positioned in front of the Thorlabs Laser Diode (808 nm, 1 W.cm^-2^ for 6 and 10 min) and irradiation began at a baseline of room. Cell viability after laser irradiation was assessed using an MTT assay method mentioned above.

### DAPI staining of Au/rGO NCs

DAPI staining was done for visualization of the condensed and fragmented nuclei of apoptotic cells treated with Au/rGO NCs in presence/absent of laser irradiation. For this purpose MCF7 cells were seeded in six-well plates containing 12 mm coverslips (2 × 10^5^ cells per well in 6 well plate) and treated with Au/rGO NCs. After 3 h of incubation, the culture medium containing un-uptake NCs was removed and a fresh medium was added. Then wells were exposed to Thorlabs Laser Diode (808 nm, 1 W.cm^−2^, 10 min) and incubated for 24 h. Untreated cells considered as negative control and the cells treated with laser alone or Au/rGO NCs alone were considered as positive control. Then each of the wells was washed with PBS, fixed by 10% formaldehyde and then cells were permeabilized by Triton X100 (10% wt) for 15 min. Finally, cells were stained with 300 ng/ml DAPI for 5 min and were visualized by a fluorescence microscope (Olympus microscope Bh2-RFCA).

### Cell cycle of Au/rGO NCs

Cell cycle assay determines the population of cells at each stage of the cell division process by DNA staining. MCF7 cells were seeded in 6 well plates (2 × 10^5^ cells per well in 6 well plates) and incubated for 24 h to be attached. Then cells were treated with Au/rGO NCs. After 3 h of incubation, the culture medium containing un-uptake NCs was removed and a fresh medium was added. Then cells were exposed to Thorlabs Laser Diode (808 nm, 1 W.cm^-2^, 6 and 10 min) and incubated for another 24 h. Then, the cells were harvested by trypsinization and proper PBS washings. Then the cells were fixed in cold ethanol. After 72 h incubation at 4 °C, cells were washed and treated with 10 µL ribonuclease A, with the subsequent addition of propidium iodide (PI) at dark. Evaluation of fluorescence signals was done by FACS set from Beckton Dickinson Company.

## Results and discusion

### UV–vis evaluation

**Figure 1:**
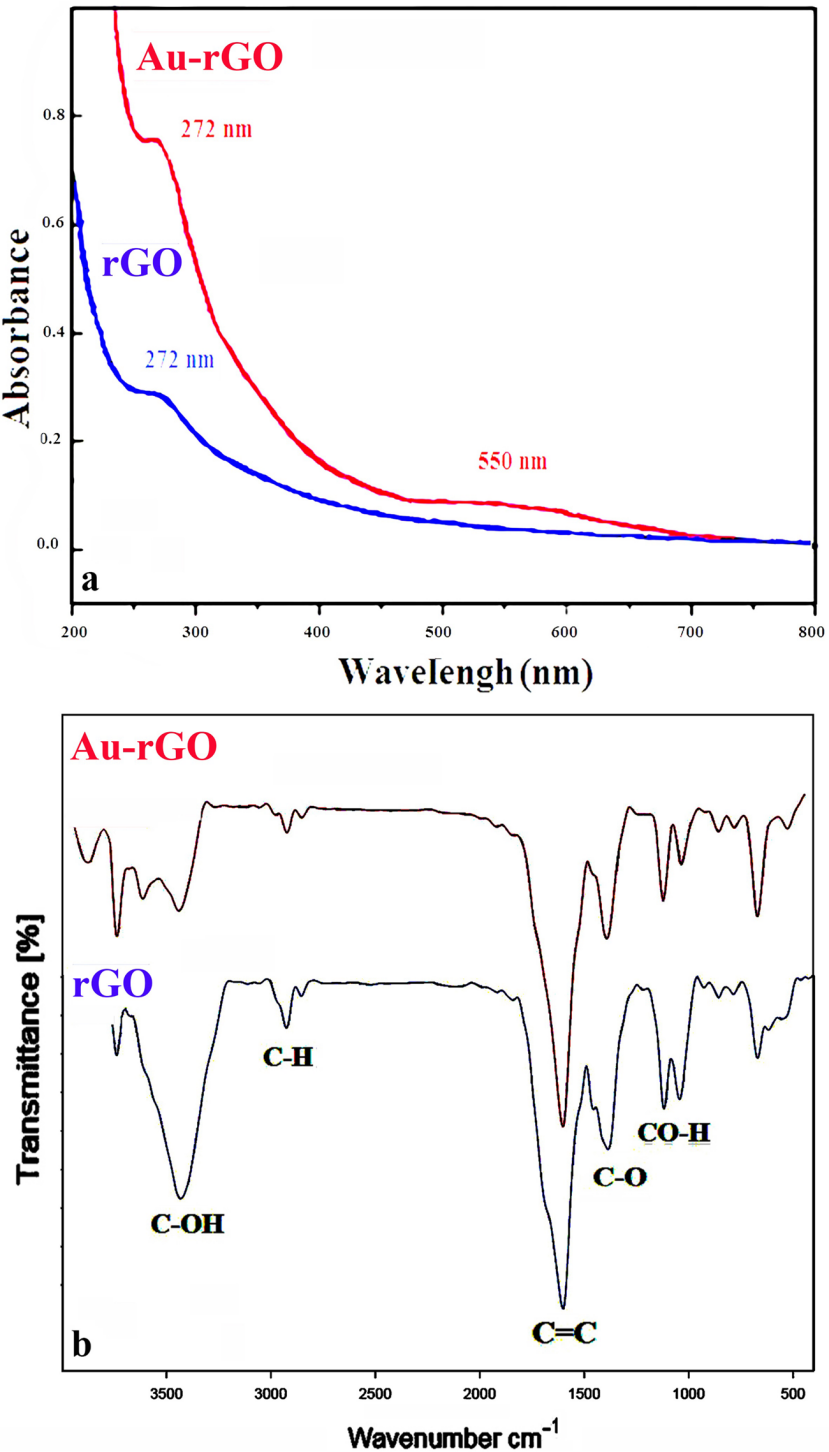
(**a**) UV–Vis spectra of rGO and rGO/Au NCs; (**b**) FTIR spectra of rGO and rGO/Au NCs.

Figure 1A shows UV–Vis spectra of rGO and Au/rGO NCs. The immobilization of Au NPs on the surface of rGO is confirmed by detecting a band at 550 nm. This signal is attributed to Au NPs' extended Surface Plasmon Resonance (SPR) band of Au NPs^35^. It is well-known that the diameter, dispersity and morphology of Au NPs affect the maximum extinction of SPR bands. The extended character of the SPR band originating from the clustering and dispersity of Au NPs. Figure 1A demonstrates the effective immobilization of Au NPs on the surface of rGO as Au/rGO NCs^24^.

### FTIR assay

The synthesis of Au NPs on rGO via *B. oleracea* extract was verified by FT-IR spectrophotometric measurement (Fig. 1B). The bands at 1043 cm^−1^, 1380 cm^−1^ and 1600 cm^−1^ are attributed to the C–O (alkoxy), C–O (carboxylic) stretching, and C=C skeletal aromatic ring vibrations, respectively. The absorption bands at 2800 and 2900 cm^−1^ attributed to the C–H stretching vibrations^36^. The wide band at 3400 cm^−1^ assigned to O–H stretching on the surface of rGO^4^**.**

### Mechanism

**Figure 2:**
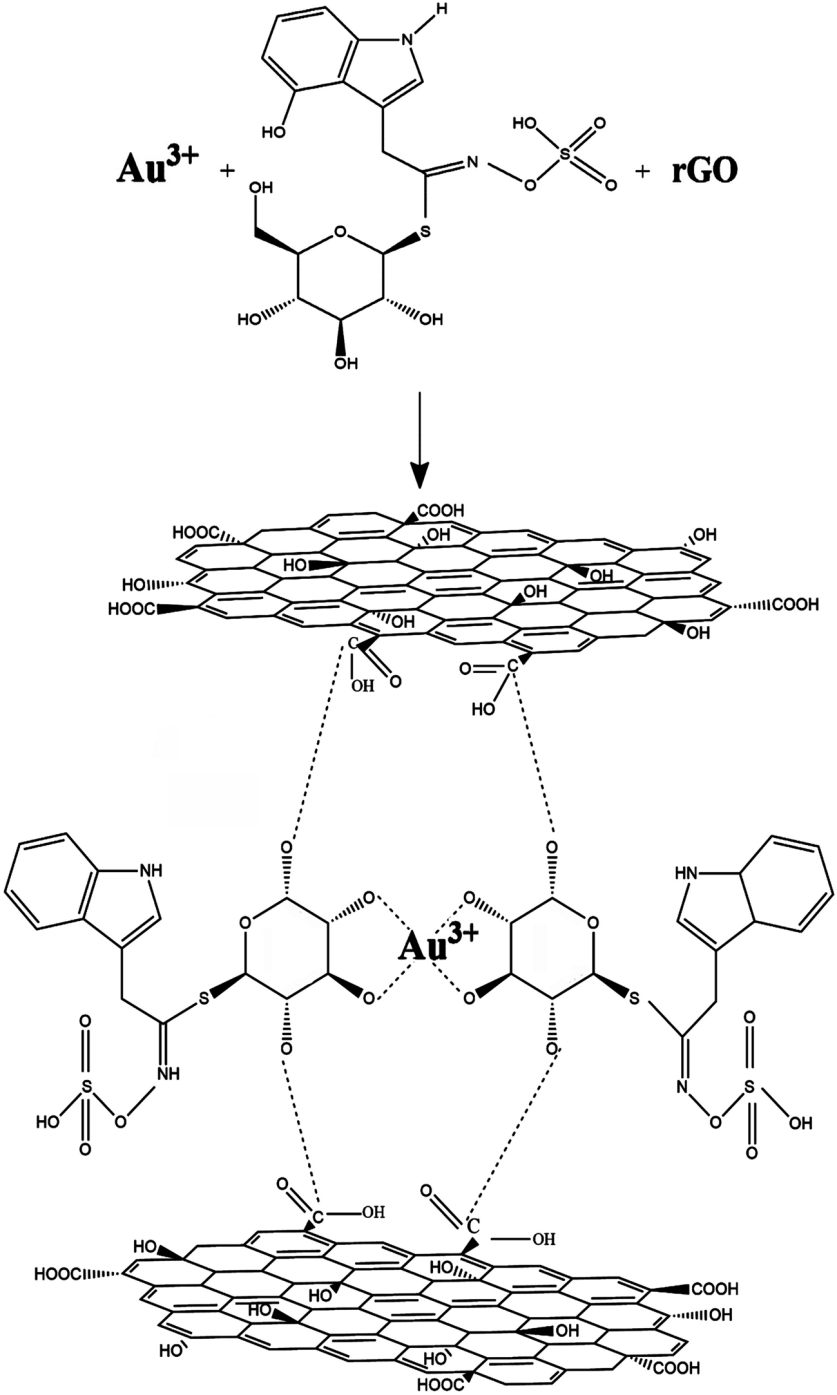
The possible mechanism for formation of rGO/Au NCs.

Analysis of the *B. oleracea* revealed the presence of 8 glucosinolates, 12 anthocyanins, 2 carotenoids and 7 phenylpropanoids^27–30^. Glucosinolate contents change among the different parts and types of *B. oleracea*. Vicas et al. found 4.89 μmol/g deionized water of total glucosinolate content in *B. oleracea*, predominated by indolyl glucosinolates (glucobrassicin, neoglucobrassicin, 4-methoxyglucobrassicin)^29^. It appears that hydroxl groups of the metabolites and their van der Waals bonding with a functional group in the surface of rGO are responsible for the chelation reaction^4,37^^.^ Glucoside segments of glucosinolates possess very strong chelation activity. It is due to the presence of hydroxyl groups in glucoside sections which can chelate the metal cations in medium (Fig. 2). In addition, glucosinolates from van der Waals bonding with such carbonyl and epoxy functional groups on the surface of rGO sheets.

### XRD patterns

**Figure 3:**
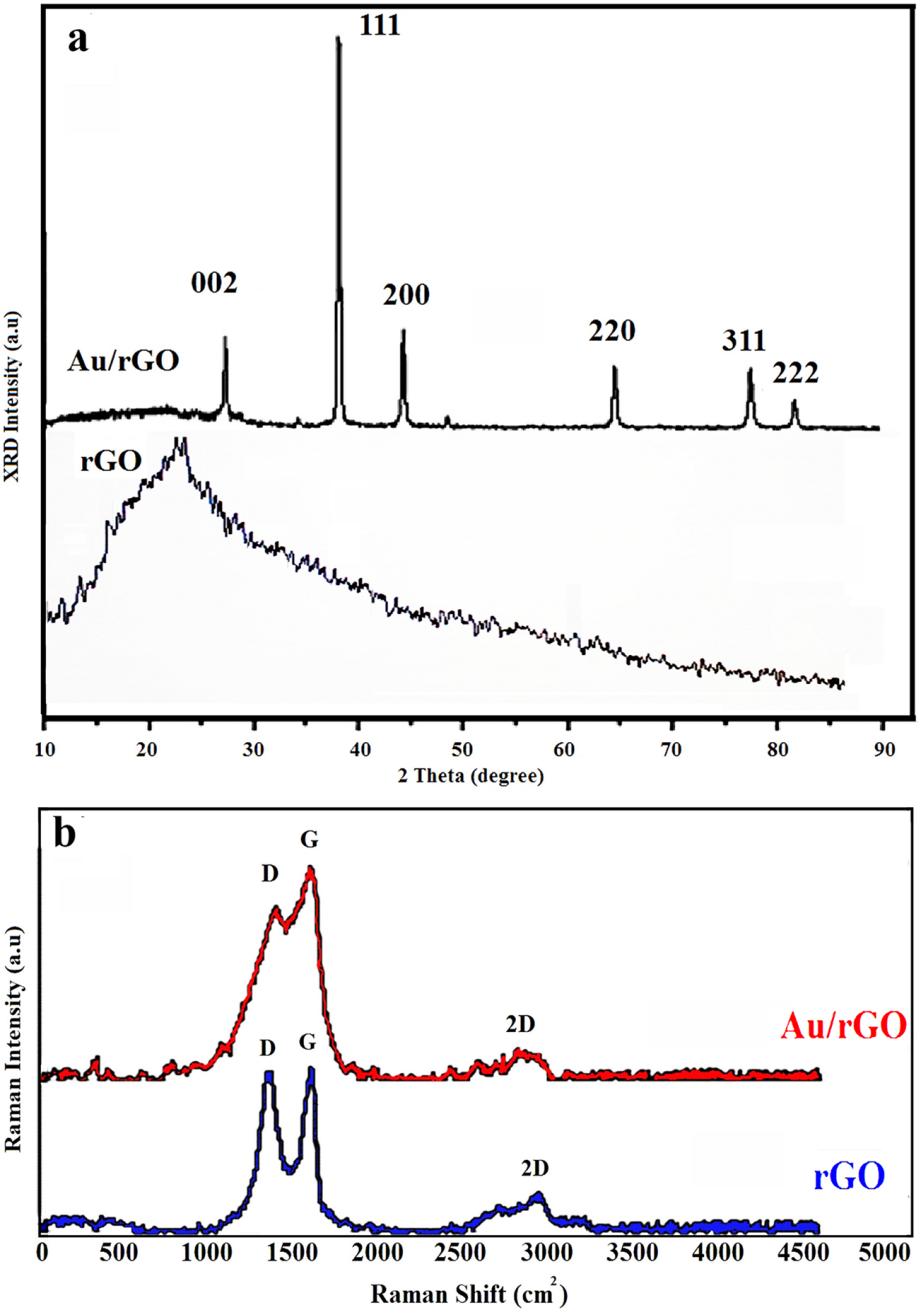
(**a**) XRD patterns of rGO and Au/rGO NCs; (**b**) Raman spectra of prepared rGO, Au/rGO NCs.

Formation of the Au NPs on rGO nanosheets was confirmed by XRD analysis as shown in Fig. 3a. The rGO samples showed a main reflection peak on 24.85° related to (002) crystallographic plane. The XRD pattern of Au/rGO NCs shows prominent peaks on 2θ values of 38.12^°^, 44.33^°^, 64.59^°^, 77.68^°^, and 81.76° which were attributed to (111), (200), (220), (311), and (222) planes of cubic crystallites of Au NPs, respectively (JCPDS card No 004–0784)^24^. In addition, the peak that appeared at 24.85° on XRD patterns of Au/rGO NCs was attributed to rGO sheets. The intense diffraction peak observed at 38.12^°^, corresponded to the crystalline Au, confirms that the NPs were composed of pure crystalline Au^35^. The crystallite size of Au NPs was calculated by Scherrer’s Eq. ^32^. (Eq. 1)

1 $$ d = k\lambda /\beta \cos \theta $$

where *K* = 0.9 is the shape factor, λ is the X-ray wavelength of Cu Kα radiation (1.54 Ǻ), θ is the Bragg diffraction angle, and β is the full width at half maximum (FWHM) of the respective diffraction peak. Based on the Scherrer equation, the calculated average size of Au NPs in the Au/rGO NCs was found to be 12.98 nm (Table 1).

**Table 1:** Shows the colloidal properties of rGO and Au NPs in the Au/rGO NCs.

Samples	D (nm)			PDI (μ_2_/T^2^)^e^	Z^f^ (mV)
	XRD^b^	SEM^c^	DLS^d^		
rGO	–	40	96.9	1	− 29.9
Au NPs^a^	12.98	12 − 18	24.69	0.87	− 7.95

^a^Au NPs in the Au/rGO NCs.

^b^Theoretical diameter calculated by Origin software based on Scherrer's equation.

^c^Particle mean diameter measured by SEM.

^d^Hydrodynamic diameter measured by DLS in water.

^e^Polydispersity index based on DLS.

^f^Zeta potential.

### Raman assay

Figure 3b depicts the Raman spectra of the synthesized rGO, and Au/rGO NCs. The important bands to consider are D, G and 2D. The D band (1200–1500 cm^-1^) is associated with the defects or vacancies present in the carbon structure and with the presence of sp^3^ carbons. G band (1500–1600 cm^−1^) is related to in-plane vibrations of sp^2^-bonded carbon atoms. In this figure, the ratio of ID/IG stands for band intensity and shows the number of disorders in graphene derivatives. The D and G bands of rGO appeared at 1360 and 1580 cm^-1^ respectively, while for Au/rGO NCs a relative shift towards lower frequencies of 1350 and 1575 cm^-1^ was observed (Fig. 3b). The ID/IG ratios for rGO and Au/rGO NCs were 0.99 and 0.81, respectively. As it is seen, the intensity ratio ID/IG of Au/rGO NC decreases considerably relative to the rGO. This fact demonstrates that the immobilized AuNPs on rGO decreases the defects on rGO sheets in the synthesized Au/rGO NCs^35^. The 2D band (2600–2700 cm^−1^) is a second-order two-phonon process that shows the number of graphene layers. Figure 3b depicts the packing of graphene layers after reduction due to decreasing of functional groups which results in the appearance of a 2D band. The presence of the 2D band indicates the formation of rGO as a multilayer instead of a monolayer^38^. Therefore, the 2D band shows the high number of graphene layers in our synthetic method.

### Physicochemical properties; FE-SEM images, EDX analysis, DLS and zeta potential

**Figure 4:**
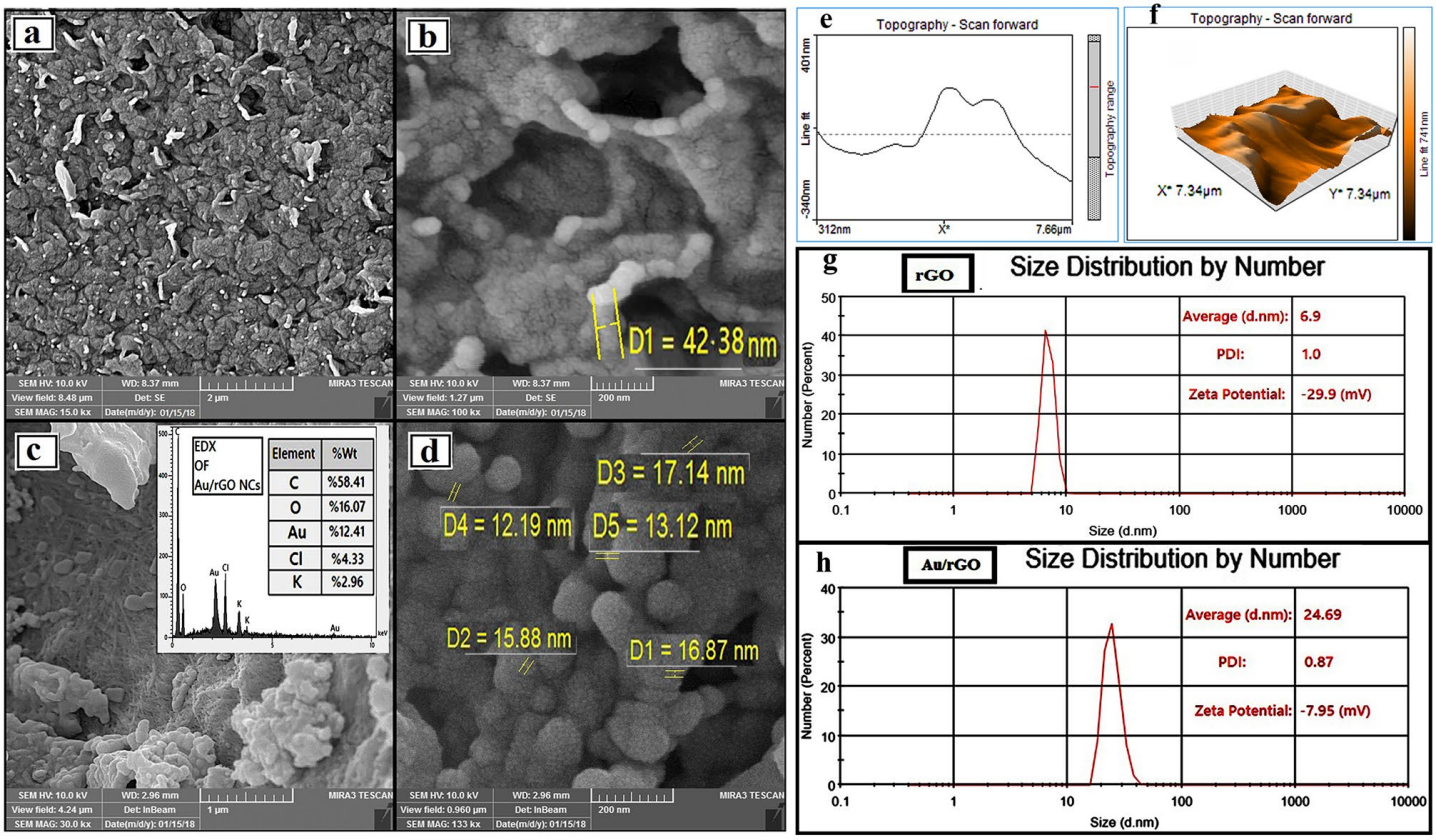
FE-SEM images of rGO (**a**, **b**), Au/rGO NCs and EDS pattern of Au/rGO NCs in insets of thumbnail (**c**, **d**); AFM image of rGO (**e**, **f**); DLS of the rGO (**g**) and Au/rGO NCs (**h**).

Figures 4 a-d indicate the FE-SEM images of rGO (Fig. 4a, b), Au/rGO NCs (Fig. 4c, d) and the EDS pattern of the Au/rGO NCs (Fig. 4c). Figure 4a shows that the rGO sheets consist of more individual sheets that are closely associated with each other. A high magnification FE-SEM image of rGO showed that the rGO sheets exist a flaky layered morphology (Fig. 4b). Generation of these sheets may originate from carbonization of the *B. oleracea biomass* through a microwave-assisted method for fabricating rGO. Therefore, the controlling of carbonization affords a tailored thickness of rGO layers^35^. The FE-SEM images of the Au/rGO NCs showed that spherical Au NPs are homogeneously immobilized on the surface of the rGO (Fig. 4c, d). The thickness of rGO layers was around 40 nm (Fig. 4b). Figure 4d demonstrates that Au NPs have sizes ranging from 12 to18 nm. As it is seen from the SEM images, the Au nanoparticles have been aggregated to some extent. So, it appears that this difference in assessing Au nanoparticles' size between XRD and SEM experiments return to the aggregation of the synthesized nanoparticles. The EDX analysis obviously depicted that C, O and Au elements are present in Au/rGO NCs (w/w %; C = 58.41%, O = 16.07%, Au = 12.41%) (Fig. 4c). In this study rGO and rGO/Au NCs were synthesized by using *B. oleracea* biomass and its extract. K and Cl have an important role in the physiological aspects of plants. For example, Potassium is the second most abundant nutrient in plant photosynthetic tissues after nitrogen^39^. In addition, previous studies with isolated chloroplasts have indicated that Cl^-^ is an essential cofactor for photosynthesis^40^. So, K and Cl may be present in the context of the plant biomass and its extract. Therefore, it appears that these elements in Fig. 4c originating from the *B. oleracea*.

As can be seen from AFM evaluation (Fig. 4e) the flaky layered nature of rGO sheets provides good contact with the flat substrate. According to Fig. 4f, also, there are some folds and bumps occurring at the surface of rGO sheets. It is because of the presence of some oxygen-containing functionalities such as carbonyl, hydroxyl exists on rGO surface^4^.

The particle size of the rGO and the Au/rGO NCs was recorded by DLS technique and average hydrodynamic diameters were 6.9 and 24.69 nm, respectively (Fig. 4g, h). The quantity of Zeta potential for the rGO and the Au/rGO NCs was approximately -29.9 mV and -7.95 mV, respectively. The decrease in negative charge of Au/rGO NCs compared to rGO proved the successful synthesis of Au NPs on rGO^41,42^. The colloidal properties of rGO and Au NPs in the Au/rGO NCs was showen in Table 1.

### Antioxidant activity

**Figure 5:**
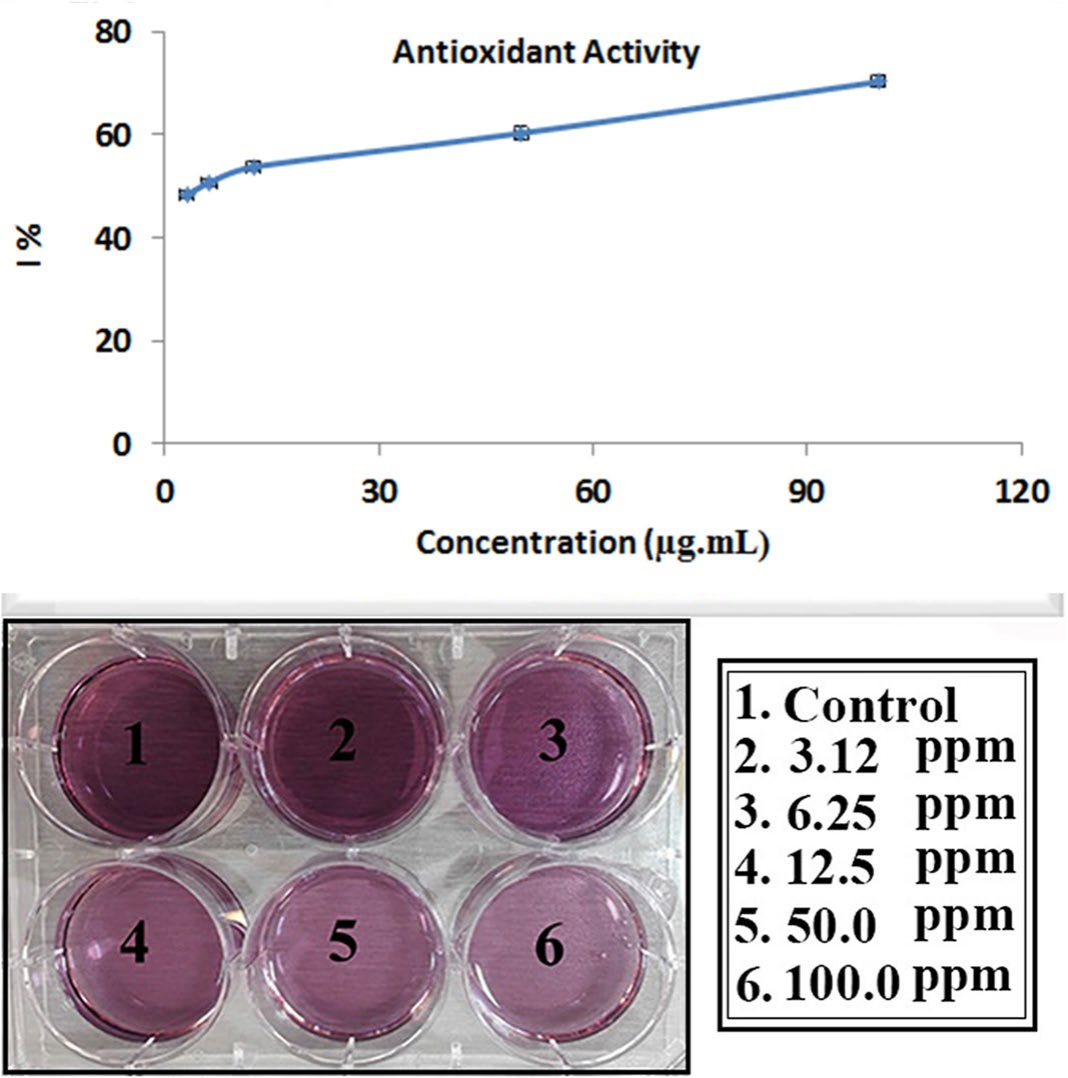
Radical scavenging activity and ROS generation of the Au/rGO NCs.

Phyto-compounds of a plant extract plays an important role as antioxidants and reacts with DPPH^34^. Graphene- based nanostructures may generate ROS at their surfaces and these radicals can oxidize the biomacromolecules of the human body^43^. Hence, it is viatle to evaluate the obtained data from the radical scavenging activity of Au/rGO NCs. In the present study, the DPPH assay method was used to investigate the antioxidant property of Au/rGO NCs. As previously mentioned, various kind of antioxidants of the extract can perform synergistically (please see section "Mechanism"). During the synthesis of the Au/rGO NCs, these phyto-compounds are pooled into the NCs. They adsorbed onto the surface of the Au/rGO NCs. With considering the high surface area to volume ratio in rGO, it appears that Au/rGO NCs show a high tendency to interact with and reduce DPPH (Fig. 5). In this survey, the obtained results did not show meaningful change in generating ROS for Au/rGO NCs in the lack of radiation sources (Fig. 5). In addition, the results of ROS production did not display any concentration change with radical generation for the Au/rGO NCs^43,44^.

### Cytotoxicity assay

**Figure 6:**
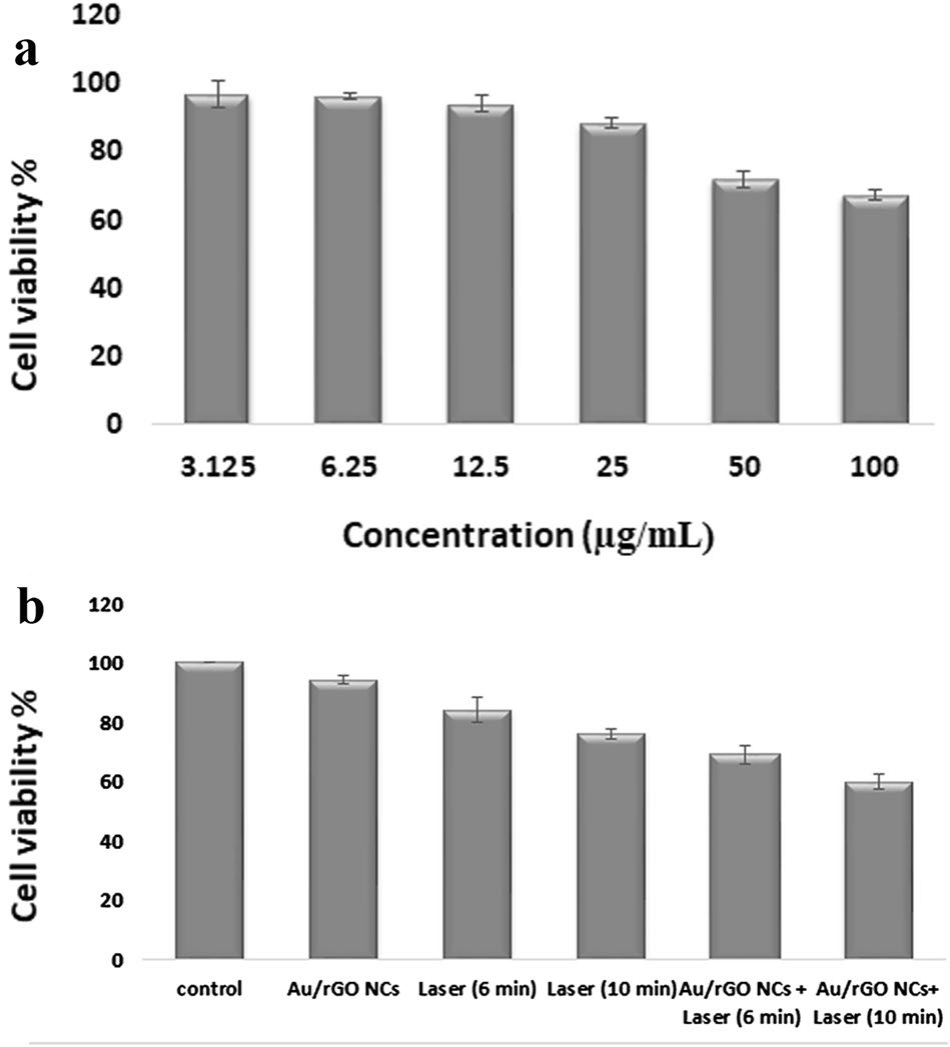
(**a**) Cell viability results of the MCF7 cells treated with Au/rGO NCs; (**b**) Cell viability of the MCF7 cells in presence and absence Au/rGO NCs and exposure NIR irradiation for 6 and 10 min.

In order to further evaluate the Au/rGO NCs potential for biomedical application, the cytotoxicity of Au/rGO NCs was evaluated to MCF7 tumor cells at different concentrations by conducting MTT assay method. The results showed that the optimum percentage of Au nanoparticles doping in rGO is up to 12.5 µg.mL^-1^, because of the non-toxicity of Au/rGO NCs to MCF7 cell lines up to this concentration. With increasing Au/rGO NCs concentration from 12.5 to 100 µg.mL^-1^, cytotoxicity has appeared. The results of Au/rGO NCs on cytotoxicity are given in Fig. 6a. Our results showed that the novel synthesized Au/rGO NCs showed very low cytotoxicity and the obtained IC50 value on MCF7 cell lines was about 110 µg.mL^-1^. In a study conducted by Saikia et al., the cytotoxicity of Au-rGO against PC3 and RWPE-1 cells were higher with an IC50 value of about 12.02 and 25.1 µg.mL^−1^^43^. Concentrations of 12.5 µg.mL^-1^ due to non-toxicity to the MCF7 cells was chosen for laser therapy studies in the present work.

### In vitro laser therapy

Figure 6b demonstrated the cell viability of MCF7 cells treated with 12.5 µg.mL^-1^ of Au/rGO NCs after laser irradiation with Thorlabs Laser Diode (808 nm, 1 W.cm^-2^ for 6 and 10 min) by MTT assay. The mortality rate of MCF7 cells treated with Au/rGO NCs after 6 and 10 min of exposure to laser irradiation was about 30 and 40%, respectively. Irradiation for 10 min in the presence Au/rGO NCs led to a significant decrease in the cell viability. While at the same condition the mortality rate of MCF7 cells exposed to only laser irradiation for 6 and 10 min without Au/rGO NCs treatment was below 20%. These results indicated that Au/rGO NCs treatment especially can improve the efficacy of laser therapy in breast cancer cells destruction.

### DAPI staining results

**Figure 7:**
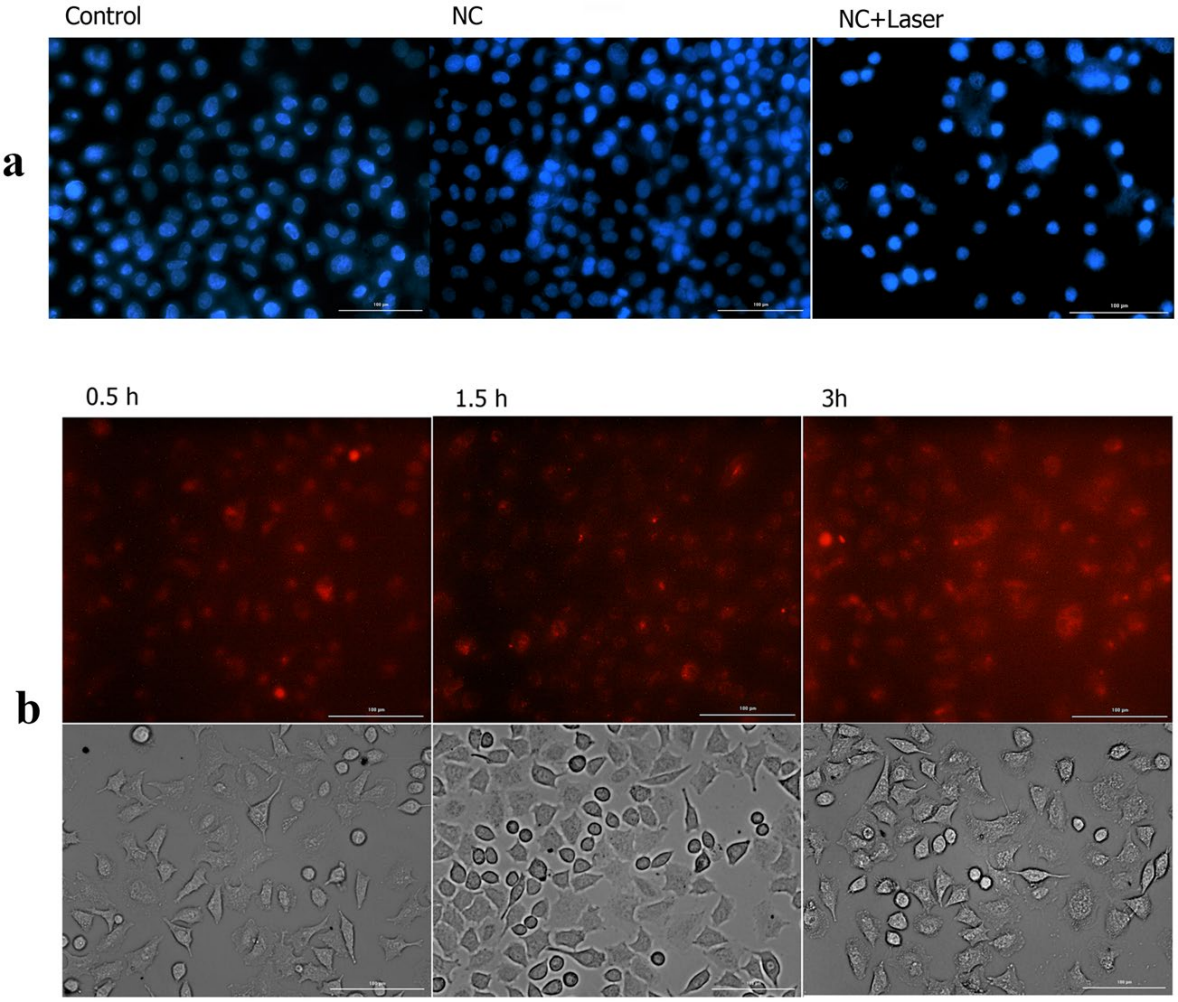
(**a**) Microscopic images of DAPI stained MCF7 cells after exposure by NIR laser irradiation for 10 min , untreated MCF7 cells (negative control), Au/rGO NCs and Au/rGO NCs + laser; (**b**) Cellular uptake of MCF7 cells lines treated with Rhodamine B-labelled Au/rGO NCs for exposure durations of 0.5, 1.5 and 3 h captured by florescent microscopy.

Degree of nuclear condensation and fragmentation of MCF7 cells was investigated via the DAPI staining approach. The control MCF7 cells, showed normal nuclei and no apoptosis or necrosis of cancer cells nucleus was observed. MCF7 cells treated by Au/rGO NCs showed relatively low toxicity. But when the cells were treated with Au/rGO NCs with laser irradiation, an obvious increase in nuclear condensation was observed and the cell viability was significantly decreased (Fig. 7a).

### Cell uptake of the Au/rGO

The cellular uptake and intracellular distribution of Au/rGO NCs by breast cancer cells MCF7 evaluated with fluorescent microscopy. Figure 7b shows the respective cellular uptake images of Au/rGO NCs captured by fluorescent microscopy. Due to the increase in fluorescence intensity with incubation time, the possibility was strengthened that the cellular uptake of Au/rGO NCs maybe via endocytic pathway^45^. Au/rGO NCs distributed throughout the cytoplasm as the intracellular red fluorescence was mainly emitted from that region. Cellular uptake and accumulation of Au/rGO NCs would efficiently induce the obvious intracellular photothermal conversion for cancer PTT and PDT treatment^46^.

### Cell cycle arrest results

**Figure 8:**
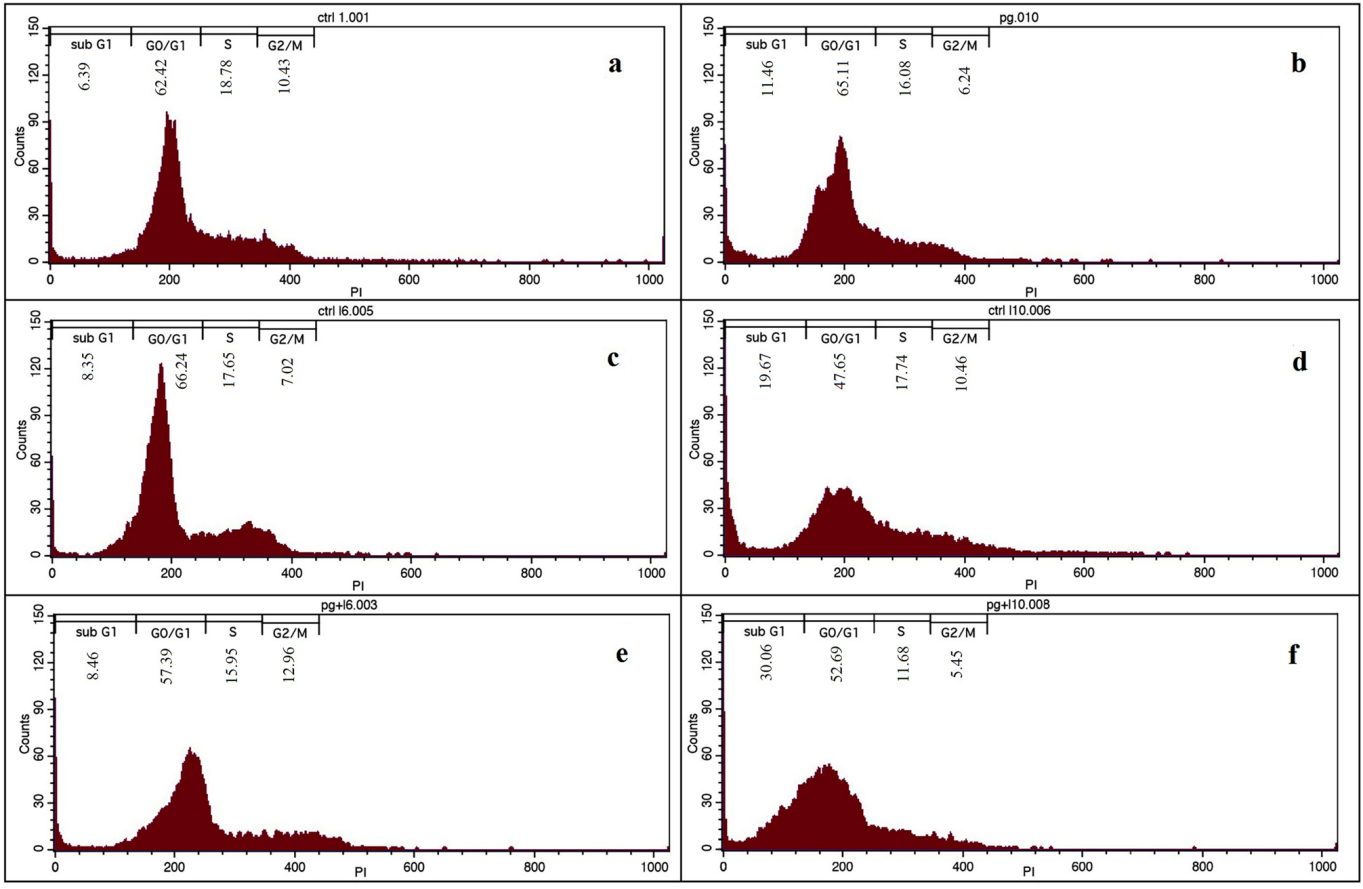
Cell cycle arrest quantitative results obtained by flow cytometry, negative control (untreated cells) (**a**), cells treated by Au/rGO NCs (**b**), cells irradiated for 6 min (**c**) 10 min (**d**) (positive control), cells treated by Au/rGO NCs irradiated for 6 min (**e**) and 10 min (**f**).

For analyzing DNA content as an index of cell generating, reagents including DNA dye such as propidium iodide (PI) are used. In this study, the cell cycle results showed that the cells treated with only laser irradiation (808 nm, 1 W/cm^2^, 6 min) or only Au/rGO NCs did not show a significant change in the cell cycle pattern of MCF7 cells compared to untreated cells (Fig. 8). But by increasing the laser irradiation time to 10 min, a change in cell cycle pattern of MCF7 cells was observed and the percentage of cells in sub G1(as a marker for apoptotic cells) was increased(˜19%). In treatment groups that received a combination of Au/rGO NCs and laser irradiations for 10 min more obvious change in cell cycle pattern was observed and a large number of cells were transferred to the sub G1phases. Observing the highest sub G1 (˜30%) cell arresting can also prove that by the combination of Au/rGO NCs and laser irradiations, apoptotic is a dominant pathway of cell death.

## Conclusions

For the first time in this study, a simple one-step method was performed by using *B. oleracea* biomass for fabricating rGO successfully. The synthesis is easy, fast, cost-effective and based on a totally green procedure. Furthermore, gold nanoparticles immobilized on the surface of synthesized rGO for fabricating Au/rGO NCs. This process was, also, performed via an eco-friendly route of fabrication using *B. oleracea* extract as a reducing agent. A mechanism for synthetic steps of Au/rGO NCs was suggested based on experiments and other results which ontained during this study. The synergistic properties of both rGO and Au/rGO NCs demonstrate an increased potential for applications in NIR photothermal energy conversion capability. Therefore, we could successfully induce a photothermal effect against breast cancer at ultralow concentration. According to cellular uptake study, Au/rGO NCs were uptaked by MCF7 cells after half-hour of treatment. DAPI staining results revealed that when the cells were treated with Au/rGO NCs with laser irradiation, an obvious increase in nuclear condensation and defragmentation was observed and the cell viability was significantly decreased. Also, cell cycle arrest showed that MCF7 cells treatment with Au/rGO NCs with laser irradiation for 10 min led to sub G1 cell cycle arrest which is dominant in apoptosis cell death pathways. Therefore the PTT of human MCF7 carcinoma cell lines after treatment with Au/rGO NCs (12.5 µg/L) and exposure to NIR laser with a power of 1 W.cm^-2^ resulted in the inhibition of MCF7 cells growth by inducing of apoptosis. Such high efficacy may be owing to the synergetic impact of rGO and Au NPs. Finally, the green synthesized Au/rGO NCs act as novel photosynthesizer agent for PTT of MCF7 cancer cells with promising application in the field of nano-biomedicene.
